# General Paralysis*Read before the Cincinnati Academy of Medicine, October 23, 1882.

**Published:** 1883-02

**Authors:** Philip Zenner


GENERAL PARALYSIS.* By PHILIP ZENNER, A. M., M.D.

To the majority of physicians, insanity, in many of its aspects, is a sealed book. Their intimate relations with people of all ranks and all grades of intelligence, sharpens their natural powers of

* Read before the Cincinnati Academy of Medicine, October 23, 1882.

discrimination, and enables them to say in this or that case, there is a departure from the normal; there is a degree of mental unsoundness. But, as regards the different types of insanity, their development, modes of manifestation, and pathological substrata, they are usually not informed. Nor can it be demanded of the busy practitioner, that he should possess that special knowledge, whose attainment demands of the specialist years of diligent study, the services of the latter being always at his call. But there is one form of insanity, which attacks the most valuable members of society, able-bodied, hard-working, intelligent men, which comes on insidiously, and threatens the deepest interests of the family and society, that every physician should know, and know well. It is that usually termed general paralysis of the insane, or dementia paralytica.

To the intelligent being, there is nothing more interesting than observing the development of intellect; to see it budding in the crowing infant and prattling child, unfolding its powers in the youth whose increasing knowledge and growing mastery over self display his strengthening character, or achieving its wonderful conquests in the realms of industry, science or art in the adult man. But the study of this disease brings before us quite an opposite development. We will have to do with the decay, the saddest chapter in the history of intellect. In its retrograde course, it will not infrequently remind us of the intellectual status of the infant and the child.

Within the limits of a brief article, we will necessarily be enabled to do but partial justice to our subject. Many points we must pass in complete silence. Some, even of importance, can be barely mentioned. Again, others we will try to enter into fully, especially such as are of great practical value, or whose thorough comprehension may be necessary for a proper understanding of the disease.

General paralysis is essentially an affection of the anterior portion of the cerebrum, of that part which the study of comparative anatomy and anthropology indicates to be the seat of intelligence, and which modern experimental investigations indicate to contain the motor centers. And it is morbid manifestations of intelligence and of the motor functions, occurring simultaneously and progressing co-relatively, that constitute the essential features of this disease.

The pathological anatomy consists of changes in the membranes of the brain, usually most marked in the anterior portions, and changes in the cortex and subcortical regions, affecting chiefly the anterior cerebral convolutions. The minute changes in the brain substance are still matters of controversy. In this place, where we wish to enter into important practical considerations only, we will not complicate our study with the many theories and facts in this relation, but content ourselves with the knowledge that the result of the pathological processes is atrophy of the brain.*

With the question of causation we will also be brief. The disease occurs chiefly in men, only in adults, and at the prime of life. Hereditary influences, or other predisposition to cerebral disease, as former disease or structural defect, seem here to play little or no part. It appears to be caused directly by the injurious influences with which one has to contend in the struggle for existence. We find the disease usually in those who have just been performing unusually prolonged and difficult intellectual work. But, as mental overwork scarcely ever has that disastrous influence exerted by passion or other undue strain of the affective life, so we can seldom, if ever, attribute the production of the disease to intellectual strain alone; but will find also a history of unusual excitement, of anxiety, or, perhaps most frequently, of great disappointments. In proof of the overwhelming weight of these factors, the disease is especially rife after great financial crises or political revolutions. The importance and frequency of these causes are universally recognized. Some believe that alcoholism, excessive venery and syphilis (?) are frequently causes of the disease. It occurs with different degrees of frequency in different nationalities. In the highlands of Scotland and Ireland its

---

* In advanced stages of the disease, the atrophy is very apparent. Meynert has further established, by weighing separate portions of the brain in a large number of instances, that the atrophy is most marked in the anterior cerebral convolutions. The pathological processes that lead to such atrophy are still subjects of discussion, and far from being established. We will mention two of the most plausible theories. The first, that of Lubinoff, is that there is primarily a hyperæmia, due to disturbances of the sympathetic nervous system, this hyperæmia causing a mechanical obstruction to the lymph circulation, and thence leading to further changes. There is much that speaks for the correctness of this view. The second theory referred to is that the disease is an interstitial encephalitis.

occurrence is rare. Steinen declared that it is hardly known in Java. Its frequency has been ascertained in some colored races. Altogether, it can scarcely be doubted that it is most frequently found in the most highly civilized countries, just as it is most frequently found in highly intellectual individuals.*

We come now to the symptomatology, the most important part of our subject.

He who has previously been active and diligent in his business transactions, is observed to become listless in his manner, and careless of his affairs. Instead of going to his place of business, he lies listlessly about the house, or he appears to be absent-minded, forgets his hat and cane, or perhaps takes an entirely purposeless drive. It is usually said that at this time his memory is already weaker, as he cannot recall the events of the day, can not tell what he has just eaten at a meal, etc. But we would little comprehend his mental condition, should we attribute such occurrences to mere impairment of memory. The latter, as ordinarily understood, is as yet unaffected. The events of a past time can be as readily recalled as ever before. It is only of present occurrences that he takes little cognizance and has no recollection. It is because his power of attention is beginning to fail, that he notices only slightly things about him, and therefore does not remember them. In this disease, the highest faculties of intellect are first to fail. Memory can scarcely be called one of its highest faculties. See the many gifted men with but ordinary memories, and occasionally a but poorly gifted individual with marvellous powers of recollection. But the powers of attention, concentration, abstraction, are most marked in the highest intellects, and when the mind is in its best condition. Our patient, in whom these higher attributes are failing, is in the condition of a day dreamer. He but follows the association of ideas as they uncontrolledly flit through his consciousness. He has lost the power of attention which brings things singly into consciousness, and makes them to be easily remembered.

With the failure of the power of attention is early associated

* In a paper read at the last International Medical Congress, on the "Race Relationship of General Paralysis," the author suggests that the use of the potato for a diet is perhaps the cause of the infrequency of the disease in Ireland!

an impairment of the judgment. He mismanages his business. He entertains senseless projects. And soon those delusions begin to appear, which, to the laity, have always been the most noticeable and marvellous features of this disease, which the French have termed *délire des grandeurs*, the delirium of grandeur. Faint indications of this kind may be early detected, but the outspoken delusions are only present when the judgment is entirely in abeyance. At this time, anything that is desirable seems possible to him. If the patient be a female, delusions are most likely to be on the subject of dress. Though she be attired in rags, she speaks proudly of her silks and satins, of her jewels and precious stones. In man, the delusions are usually of riches, of power or glory. In his squalid den he gratuitously dispenses millions, and proclaims himself a prince or a sovereign. And, further, on account of his low state of intellectuality, his delusions are constantly changing.

The grandiose character of the delusions of this disease has been attributed to irritation of the cortical hemispheres. But, it seems to me, an explanation based upon psychological data would be clearer, and, perhaps, more correct.

In the great majority of cases, a feeling of elation and well being is dominant during the whole course of the disease. The patient never has any feeling of illness, but seems to overflow with a sense of health and power. Hutchinson has well insisted upon the fact that temperament is usually but the expression of the state of physical health or bodily comfort. In cases of strutting health, with the prevailing feeling of well-being, we will ordinarily find a sanguine temperament, while a melancholic is perhaps the result of past disease or present suffering. Nowhere else, so much as in general paralysis, does this feeling of physical health so domineer every thought and utterance. It renders the disposition an exceedingly joyous one, and it makes of every vagrant fancy an uttered delusion. The infant, with its sense of muscular power, stretches his hand toward the moon in order to grasp it. The paralytic, who has scarcely more judgment than the infant, and whose weakened intellect, unobservant of external things, dwells only on the *ego*, in his sense of health and power believes everything belongs to him, that everything is possible to him.

But in the later stages of the disease, when a smile or laughing face is nearly always present, and the most absurd delusions the only utterance, we must not suppose these to express an active joyousness or ambitious projects. He has arrived at that stage where life is scarcely more than animal life, and actions and words scarcely more than reflex actions. We have all seen how happy a mother is when she for the first time sees her infant's smile in response to her own. She thinks she sees a happy soul behind that smile, and knows not that it was merely the reflex of her own act. In our patient, in this last stage of the disease, his smile scarcely expresses a deeper feeling than the infant's. His face is only a mirror of what is passing about him. If you smile before him, his face broadens in laughter; if you assume a mournful countenance, he breaks into tears. His uttered delusions are but the remembered words and phrases of a former life.

Coincident with the impairment of the intellect, the emotions and the will become equally affected. As the power of observing external things and the judgment become weaker, the higher kinds of feeling, those which give tone to our character, the æsthetic, the ethical, etc., become fainter, and only the lower, the transitory, those excited by sensuous perceptions, arise in the consciousness. The will yields equally. Actions become impulsive, responding to the fleeting emotions, the controlling influences, which give their determined and purposive character, being lost. We can thus easily account for the impulsive and irresponsible actions of the paralytic, and have only to add that the intensity of his emotions and the energy of his actions have also become much enfeebled.

We have now reviewed the general changes in the intellect and character of the paralytic in their general decay. We have attempted to view them from the higher standpoint of their psychological causes, as learned from the natural development and decay of mind. We have given no full description of his mental condition; of the many delusions he may entertain; of the mental weakness displayed, as much in the easy manner in which he is diverted from his schemes, as in the schemes themselves; in his utter incapacity to judge of the acts and motives of others, as well as his own, and, further, of his defective moral sensibility, and of his apparently motiveless actions. We cannot pretend to give a full description of this ever-varying picture. To do that it would be necessary to mention all the possible conclusions of a weakening judgment, all the feelings of a narrowed egotistical existence, and all the actions of a wavering mind. But, where it exists, the competent observer will be able to recognize the picture of mental weakness. For he, who has learned to know the springs of human actions, who knows something of the capabilities of the human mind, will be able to judge whether the performances of the individual correspond to the ability with which he has been hitherto credited, whether his actions are prompted by such motives as in a normal mind.

We come now to the second set of symptoms, the motor phenomena of this disease. These are of great interest for they recall and corroborate facts gained by experimental investigation. The paralyses are cortical in their origin. Experiment has shown that cortical lesions leave but very slight paralyses, which are chiefly manifested in complex movements. This is the exact character of the paralyses of this disease. They are mostly not readily observable, and are brought out by complex movements requiring the delicate play of muscles. The paralyses usually observed are those of the facial muscles, dilatation of the pupil (usually on the same side as affection of the facial, and that generally on the right side), and tremor of the lips and tongue. Of these we will again speak when on the subject of diagnosis.

We have said that the above are the usual paralytic manifestations of general paralysis. We might add they are in the great majority of cases the only paralyses due to the cerebral lesions. But—and here we are approaching the most inexplicable part of our subject—in very many cases of this disease there are spinal as well as cerebral lesions. It is a striking fact that in the most common of the organic diseases or the cord, locomotor ataxy, cerebral symptoms due to lesions of the optic, third, sixth, and other nerves are often present, and in this, the most common organic disease of the brain, spinal lesions are frequently found.

For the connection of the spinal with the cerebral disease, as in the other case the appearance of the cerebral symptoms with spinal disease, there has as yet been no satisfactory explanation.*

The spinal symptoms are mainly those of paraplegia. The impaired gait may be like that of lotomotor ataxy, due to incoordination of movements, or the spastic gait of spastic spinal paralysis, or merely a weakness of the muscles not characterized by either of the above types. The pathological anatomy is sometimes that of typical locomotor ataxy, sclerosis of the posterior columns. Sometimes myelitis of the antero-lateral columns.† In cases with the symptoms and pathological changes of locomotor ataxy, the cerebral symptoms of that disease, myosis, paralysis of the occular muscles, etc., are often present.

The spinal symptoms may precede those of mental derangement, but more frequently they occur later. Westphal, who discovered that the absence of the patellar tendon reflex is nearly always a sign of disease of the posterior columns of the cord, has enabled us to detect spinal lesions even when the symptoms of such disease are not present. He has thereby been enabled to determine that spinal disease occurs more frequently in general paralysis than is usually supposed, and has proved on the post-mortem table the correctness of the diagnosis of spinal disease where, during life, there was no spinal symptom.

Before concluding the subject of symptomatology we will mention a few facts, exceedingly important in themselves, but of which our limited time will allow but the mere mention : that is the occasional occurrence of epileptic or apoplectic attacks, and the not infrequent outbreak of acute maniacal excitement.

---

* The explanation that when the posterior fasciculi are affected, there has been ascending disease from the cord, when the lateral columns are affected, descending disease from the brain to the cord, is not tenable. There has been no anatomical evidence of such a continuity of disease, and clinical histories are often not in accordance with such a development.

† In the last number of the *Archiv, f, Psychiatrie und Nerven krankheiten,* Zachler reported a case with the ordinary manifestations of what has been termed spastic spinal paralysis, paresis, muscular rigidity, exaggerated tendon reflexes, etc., in the lower extremities, in which he fonnd no changes in the cord. Hence, he has clalmed that these symptoms may be due to cerebral rather than spinal diseases. Although the set of symptoms just mentioned which Erb and Charcot have attributed to disease of the pyramidal tracts in the cord have not yet with certainty been associated with any exact pathological changes, yet in the great majority of such cases, where post-mortem examinations have been made, very decided spinal lesions have been found. So that, notwithstanding the above mentioned case, I believe, we need not hesitate to assign that complex of symptoms to spinal, and not cerebral lesions.

The occurrence of peculiar disturbances of vision, first noted by Fürstner, and since then by a few others, and the efforts to bring them in relation with certain changes in the cortex, have an interest for the physiologist, rather than any clinical importance.

We just now enter into that part of our subject, practically so important, the diagnosis of the disease.

When the patient has become a maniac, when his speech consists of mere utterances of his delusions which amuse even a child, the physician is no longer called upon for a diagnosis, though even then mistakes of a kind to be later mentioned are sometimes made. The family no longer needs his advice to avert financial ruin or personal calamity, and the patient has probably been removed for safe keeping to the asylum of the alienist. It is in the inception of the disease when the weakening judgment, the germinating delusions of the one still considered by society as a responsible agent is leading him into ruinous enterprises and senseless extravagancies, possibly to be attended by an outbreak of madness which threatens human life, that the ready perception of the physician, a correct diagnosis proves of inestimable value. And at this time a correct diagnosis is peculiarly difficult.

In the beginning the changes are usually slight such as are only noticeable to those intimately acquainted with the patient. There may be some change in his disposition, but what is especially noticeable is that he does not observe and take interest in things as before, neglects his own affairs. Already there is some impairment of the intellectual faculties. But the examiner must be pardoned if he cannot yet detect a slight defect in one whose mental powers equal, and possibly surpass, his own ; for, as we have already stated, it is the gifted, the intellectual, who are most frequently affected. At least the practitioner will require all his acuteness to enable him to form a correct estimate of the mental condition of his patient at this time. He must rely partly on those who know the patient intimately, as to changes in his mental status. He must observe whether there is an apparent spirit of boastfulness, not formerly present (the first indication of the delirium of grandeur) ; try to determine whether the patient can perform higher intellectual tasks, mathematical, conversational, etc., as well as before, test patient's memory and reasoning powers, in short, test patient's intellect in every possible direction. If the disease be already well advanced his task will be easy, in the beginning it may be beyond his power. At the same time he must look for motor symptoms, differences in the pupils, paresis of facial muscles, tremor of lips and tongue, or difficulties of speech.

If these and the mental symptom are observed (and fortunately for the diagnosis the two, even in the beginning, are usually found together) the diagnosis, even in the inception of the disease, is established with a high degree of certainty.

The paralytic symptoms, as before mentioned, are very slight and may be difficult to detect. If you will permit we will dwell briefly on the best methods of making such slight manifestations cognizable. To detect slight differences in the pupils let patient look at some surface evenly lighted, as the whitened ceiling, and the observer stand in such a position as not to shade either eye. If the facial paralysis is not observed in the drooping of one corner of the mouth, or the less prominence of the naso-labial fold on one side where the face is in a state of rest, slight changes of this kind may become more distinct after the patient has spoken for some time; or may be made apparent through his squeezing the hand of the physician, when the involuntary facial expression thus elicited will reveal slight differences in the muscular innervation of the two sides of the face.

The ordinary modes of detecting facial paralysis, by the patient exposing his teeth or laughing, are such coarse movements that they conceal rather than reveal the slight motor defects. The tremor of the lips and tongue can also be best detected after the patient has been speaking for some time.

But the best test of slight changes in the speech-making organs, and one of the most reliable of the diagnostic symptoms, is the affection of speech. We do not find the scanning speech so characteristic of multiple sclerosis, but one of a different character. There is a kind of stumbling in his articulation. Words are brought out with difficulty, and are frequently cut short, letters or entire sylables being clipped off.

We repeat, such slight paralyses are characteristic of general paralysis. Only at a late period does the tremor of lips and tongue sometimes become excessive, the speech become altogether unarticulate, or the facial paralysis very decided. As regards the other motor symptoms, of spinal origin, they will seldom aid us to make a diagnosis of general paralysis.

Let us consider the distinctive character of the delusions of this disease. The *délire des grandeurs* occurs in other kinds of insanity, but there is something distinctive of it as here manifested. In other forms of insanity, where the delusions of greatness are present (excepting in some forms of dementia, kindred to general paralysis, to which we will later refer) the delusions have a certain logical basis, and are more or less permanently in one form. If one believes himself to be a prince, that delusion is based upon some erroneous conceptions, which have become a part of his mental existence, and will remain steadfastly with him. But in our case, as already stated, those delusions result from an entire absence of judgment. They are the mere expression of a vagrant fancy. They are characterized by their senselessness and their variability.

Complications which may make the disease difficult of recognition are the occurrence of apoplectic or epileptic seizures, or an outbreak of acute mania. Sometimes the disease is ushered in by one of these conditions. In that case a diagnosis is, as yet, impossible (though in some cases, even during the period of maniacal excitement, indications of mental weakness or the presence of the peculiar paralysis will reveal the true condition.) If such a complication occurs during the course of the disease the diagnosis must be based on the previous history.

We must now speak of the diagnosis of the fully developed disease. First in importance and frequency, among the diseases which may be confounded with it, is alcoholism. Chronic alcoholism may produce all the symptoms of general paralysis. Or the two diseases are not infrequently present at the same time. In cases of chronic alcoholism with symptoms of general paralysis we must abide by the rule so often imposed upon us in obscure cases, that is, allow of a certain lapse of time before making a diagnosis. The importance of this rule in such cases cannot be over estimated. For want of it mistakes are only too frequently made.

A second class of cases which may be mistaken for general paralysis is that where coarse lesions of the brain, most frequently due to hæmorrhage or embolism, are followed by dementia. In such the paralyses are usually more decided than in general paralyses, and point to a focal lesion. But the history will settle the diagnosis.

There is another class of cases, but a very rare one, in which a correct diagnosis is far more difficult; that is, cases of localized meningitis (usually primarily pachymeningitis, perhaps hæmorrhagic) occurring especially in the neighborhood of the central convolutions. That cases of this kind are often classed under general paralysis cannot be doubted. The diagnosis is often impossible. The course of the disease will alone (but not always) be able to clear it.

Lastly, we will only mention multiple sclerosis and cerebral syphilis, instances of which diseases being sometimes exceedingly difficult to distinguish from general paralysis. We must look for special symptoms of these diseases, and if these will not determine their character, we must again rely upon the course of the disease.

We have frequently mentioned the "course of the disease." This is clinically of the greatest importance. We know the disease rather by its course than its symptoms. The symptoms may be found in various other conditions, but the disease has a particular course peculiar to itself. Its average duration is three years. It almost invariably terminates fatally. Its duration is usually longer in woman than in man, longer in cases of a melancholic type than in others; and in some cases in which there are periods of improvement or apparent recovery its duration is indefinite. There is usually a gradual progress in all the symptoms, mental and motor, from bad to worse. Sometimes there are sudden and rapid changes, as an apoplectic attack followed by a considerable increase of the dementia; sometimes an improvement of indefinite duration. Death is sometimes caused by a gradual failing of all the bodily functions; more frequently it is caused by an apoplectic attack, decubitus, pneumonia, tuberculosis, or disease of the bladder or kidneys.

In the preceding description we have given the symptoms of the usual or classical type of the disease. Other symptoms may be present, producing quite a different appearance from the ordinary picture. Sometimes instead of the usual slow manner of thinking and acting, there is a very flood of ideas, an unusual vivacity in thought and manner. Not infrequently there are outbreaks of maniacal excitement, when, it must never be forgotten, the patient becomes dangerous to his surroundings. Again we meet with conditions of mental depression. There are cases in which a depressed hypochondriacal state of mind marks the whole duration of the disease. In some instances delusions, usually so characteristic of this disease, are never detected. Hallucinations may or may not be apparent in a particular case. Spasmodic muscular affections complicate the history in some instances.

And so we might add other symptoms which give peculiar appearances in certain cases. But, and this is the important part which cannot be too much emphasized, whatever the clinical manifestation presented, the diagnosis is based upon the impairment of the intellectual faculties, and the peculiar paralyses, and is confirmed by the course of the disease.

The following cases are very briefly described to illustrate the preceding remarks. Cases illustrative of the incipient stages of the disease would be more valuable, but the material is lacking. For the opportunity to examine these patients and their histories, I am indebted to Dr. Harmon, of Longview Asylum:

T. D., age thirty-four, bank clerk; duration of disease, two years; excessive venery, also addicted to excessive use of alcohol. The first history of mental disorder is that of delusions of various kinds. His speech was soon observed to be slow and hesitating, his talk foolish, did not recognize his surroundings, believed himself to be in a barracks. Habits filthy.

At the end of eighteen months he had an attack of durable hemiplegia. Since then more pronounced delusions, especially of wealth.

Present condition. Secondary contractions and rigidity of muscles of right arm and leg. Facial paralysis on the right side. Right pupil larger than the left. Tongue tremulous. Speech indistinct. Mind almost a blank.

In this case the diagnosis is based on the history. The dementia is the only symptom present pointing directly to general paralysis. The high degree of paralysis would, were it not for the history, lead us to exclude this disease. It is one of these rare cases in which an attack of hemiplegia, occurring in the course of this disease, is durable. The subsequent contractures lead to the inference that the disease has been complicated with a cerebral hæmorrhage. Yet purely cortical lesions have appeared in some cases to produce secondary degeneration of the pyramidal tracts, with subsequent rigidity of the paralyzed muscles. We will again call attention to the fact that the mental condition became rapidly worse after the attack of hemiplegia.

G. R., age thirty-five, farmer, married; duration ten years; cause unknown. In the beginning it was noticed that his actions appeared to be aimless, that he appeared to be always contented and happy, was always smiling. Soon observed delusions of wealth, also tremulousness of lips and tongue, and difficulty of speech.

Present condition. Paresis of facial muscles on the right side. Tremor of lips, tongue, and hands. Gait feeble. Can obtain no utterance from him. He appears to be entirely demented.

In this case there is the typical history, but the complete silence, and the absence of every facial expression, are unusual in this disease. We can only account for them by supposing complete dementia.—*Journal of Psycological Medicine.*

